# Regulation of gastric epithelial cell homeostasis by gastrin and bone morphogenetic protein signaling

**DOI:** 10.14814/phy2.12501

**Published:** 2015-08-19

**Authors:** Andrea Todisco, Maria Mao, Theresa M Keeley, Wei Ye, Linda C Samuelson, Kathryn A Eaton

**Affiliations:** 1 Department of Internal Medicine, University of Michigan Medical CenterAnn Arbor, Michigan; 2 Department of Molecular and Integrative Physiology, University of Michigan Medical CenterAnn Arbor, Michigan; 3 Laboratory Animal Medicine Unit, University of Michigan Medical CenterAnn Arbor, Michigan

**Keywords:** Cellular differentiation, cellular proliferation, chief cells, parietal cells

## Abstract

We reported that transgenic expression of the bone morphogenetic protein (BMP) signaling inhibitor noggin in the mouse stomach, leads to parietal-cell (PC) loss, expansion of transitional cells expressing markers of both mucus neck and zymogenic lineages, and to activation of proliferative mechanisms. Because these cellular changes were associated with increased levels of the hormone gastrin, we investigated if gastrin mediates the expression of the phenotypic changes of the noggin transgenic mice (NogTG mice). Three-month-old NogTG mice were crossed to gastrin-deficient (GasKO mice) to generate NogTG;GasKO mice. Morphology of the corpus of wild type, NogTG, GasKO, and NogTG;GasKO mice was analyzed by H&E staining. Distribution of PCs and zymogenic cells (ZCs) was analyzed by immunostaining for the H^+^/K^+^-ATPase and intrinsic factor (IF). Expression of the H^+^/K^+^-ATPase and IF genes and proteins were measured by QRT-PCR and western blots. Cell proliferation was assessed by immunostaining for proliferating cell nuclear antigen. The corpus of the NogTG;GasKO mice displayed a marked reduction in the number of PCs and ZCs in comparison to NogTG mice. Further, cellular proliferation was significantly lower in NogTG;GasKO mice, than in the NogTG mice. Thus, gastrin mediates the increase in gastric epithelial cell proliferation induced by inhibition of BMP signaling in vivo. Moreover, gastrin and BMP signaling exert cooperative effects on the maturation and differentiation of both the zymogenic and PC lineages. These findings contribute to a better understanding of the factors involved in the control of gastric epithelial cell homeostasis.

## Introduction

The oxyntic mucosa is a complex structure that contains several types of highly specialized cells, such as mucus pit, mucus neck, parietal, zymogenic, and endocrine cells. The mechanisms and the factors that regulate the homeostasis of the gastric epithelium have been only partially characterized.

The bone morphogenetic proteins (BMPs) are important regulators of a broad array of biological actions during both embryonic and postnatal vertebrate development (Kawabata et al. 1998; Takaku et al. 1999; van den Brink et al. 2001; Zhou et al. 2001; Haramis et al. 2004; Wen et al. 2004, [Bibr b28]; Beck et al. 2006; Itoh et al. 2006; Auclair et al. 2007; Bleuming et al. 2007; Bragdon et al. 2011; Shirai et al. 2011). BMP-2, BMP-4, and BMP-7 appear to be expressed in gastrointestinal tissues, where they have been shown to play a significant role in the regulation of cellular proliferation and differentiation (Kawabata et al. 1998; Takaku et al. 1999; van den Brink et al. 2001; Zhou et al. 2001; Haramis et al. 2004; Wen et al. 2004, [Bibr b28]; Beck et al. 2006; Itoh et al. 2006; Auclair et al. 2007; Bleuming et al. 2007; Bragdon et al. 2011; Shirai et al. 2011). The clinical relevance of these observations has been underscored by studies conducted in human tissues that have demonstrated an important role for BMP signaling in the inhibition of gastrointestinal tumor growth (Zhou et al. 2001; Wen et al. 2004, [Bibr b28]; Beck et al. 2006; Shirai et al. 2011).

The actions of the BMPs can be selectively blocked by inhibitory proteins that are expressed in tissues to modulate the level of activation of BMP signaling (Zimmerman et al. 1996; Bragdon et al. 2011). Of these, noggin, a secreted polypeptide present in several mammalian tissues, has been shown to bind to, and inhibit, the actions of BMP-2, BMP-4 and, to a lesser degree, of BMP-7 (Zimmerman et al. 1996; Kawabata et al. 1998; Haramis et al. 2004; Bragdon et al. 2011).

In a series of recent in vivo investigations, we demonstrated that inhibition of BMP signaling in the gastric epithelium causes profound aberrations in the normal mechanisms that regulate the proliferation, maturation and differentiation of several lineages of gastric epithelial cells (Shinohara et al. 2010; Takabayashi et al. 2014). In particular, we reported that the mucosa of transgenic mice expressing noggin in the fundic epithelium, exhibits the presence of increased epithelial cell proliferation, enhanced MAP/ERK kinase activation, diminished parietal-cell (PC) number, and expansion of populations of transitional cells expressing both the mucus neck cell marker GSII and the zymogenic cell (ZC) marker IF. In addition, the noggin transgenic mice display an increased number of gastrin-containing G cells and enhanced gastrin expression and release, findings that have been thought to represent a consequence of decreased PC number and hypochlorydria (Dockray 1999).

Gastrin is a well-established stimulant of gastric acid secretion and a regulator of gastrointestinal cell growth, proliferation and differentiation (Dockray 1999; Yassin 1999). In support of these findings, studies have shown that genetic ablation of the gastrin gene leads to decreased fundic mucosal thickness and to altered maturation of parietal and ECL cells (Friis-Hansen et al. 1998; Jain and Samuelson 2006; Jain et al. 2006).

Accordingly, we undertook studies to test the hypothesis that hypergastrinemia mediates the phenotypic changes that are observed in mice expressing an inhibitor of BMP signaling in the gastric mucosa.

## Methods

### Mice

H/K-noggin transgenic mice and gastrin knockout mice were previously described (Shinohara et al. 2010; Takabayashi et al. 2014). Animals were housed in the animal maintenance facility at the University of Michigan. All animal experiments were approved by the University of Michigan Animal Care and Use Committee. Experiments were conducted in 12-week-old mice.

### QRT-PCR analysis

Intrinsic factor (IF), TFF2, and H^+^/K^+^-ATPase *α*-subunit transcript levels were determined by quantitative RT-PCR (QRT-PCR) using primer sequences that were obtained from commercially available sources (SA Biosciences Corp., Frederick, MD). RNA was isolated from the corpus of WT, NogTG, GKO, and NogTG;GasKO mice aged 3 months, using TRIzol reagent (Invitrogen, Carlsbad, CA), followed by DNase treatment and purification with the RNeasy Mini kit (Qiagen, Valencia, CA). RT reactions were conducted according to previously published reports (Shinohara et al. 2010; Takabayashi et al. 2014). Reactions were performed using the Icycler (Bio-Rad, Hercules, CA) with a 20 *μ*L reaction in PCR buffer (Invitrogen) containing 2 *μ*L of cDNA (RT product), 5.5 mmol/L MgCl_2_, 100 nmol/L primers 200 nmol/L dNTPs, 0.1× SYBR Green, 10 nmol/L fluorescein, and 0.025 U Platinum Taq (Invitrogen). Expression levels were normalized to glyceraldehyde-3-phosphate dehydrogenase (GAPDH) expression. The cycle parameters were as follows: 95°C, 3 min, 40 cycles at 95°C, 60°C, and 72°C each for 30 sec and 72°C, 5 min. After amplification was completed, the samples were subjected to melt-curve analysis by increasing the temperature from 60°C to 100°C, in 0.5°C intervals every 10 sec for 80 steps to assess product purity. No signal was detected with control samples that were not treated with RT (data not shown).

### Western blots

Gastric samples obtained from the corpus of WT, NogTG, GKO and NogTG;GasKO mice aged 3 months, were homogenized on ice in 1-mL microdounce homogenizers (∼15 strokes) using 500 *μ*L of lysis buffer (Shinohara et al. 2010). The lysates were spun at 16,000 × *g* for 5 min at 4°C. Equal volumes of lysates containing 80 *μ*g of proteins were mixed with 5× electrophoresis buffer (Shinohara et al. 2010), boiled for 5 min (except for the H^+^/K^+^-ATPase *α*-subunit western blots, in which the samples were not boiled) and loaded on 10% SDS-polyacrylamide mini-gels, which were run at 200 volts for 1 h. After transfer and blocking (Jain et al. 2006) the membranes were incubated for 16-18 h at 4°C in 10 mL of TBST, 5% dry milk, containing specific antibodies recognizing IF (1:3000) (gift from Dr. David Alpers, Washington University, St. Louis, MO), and the H^+^/K^+^-ATPase *α*-subunit (1:1000) (Medical and Biological Laboratories, Nagoya, Japan). Control blots were performed using antibodies recognizing GAPDH (1:1000) (Chemicon International, Temecula, CA). At the end of the incubation periods the membranes were washed in TBST for 30 min at room temperature and then incubated for 1 h in TBST, 5% dry milk, containing either protein A, directly conjugated to horseradish peroxidase (HRP) (Amersham Life Science, Arlington Heights, IL) (1:2500) or an HRP-conjugated antimouse secondary antibody (1:2000) (Cell Signaling, Beverly, MA) for the H^+^/K^+^-ATPase *α*-subunit and GAPDH blots. The membranes were washed in TBST for 30 min at room temperature and then exposed to the Amersham ECL detection system according to the manufacturer’s instructions.

### Histochemical analysis

Stomachs were dissected, opened along the greater curvature, pinned flat on dental wax, and fixed in 4% paraformaldehyde. Paraffin sections (3–5 *μ*m) were immunostained, depending on the experiment, with the anti-H^+^/K^+^-ATPase *α*-subunit antibody (1:500), the anti-IF antibody (1:2000), and the anti-TFF2 antibody (1:200) (Abcam, Cambridge, MA). In additional experiments, the sections were incubated with either an antiproliferating cell nuclear antigen (PCNA) antibody (1:400) (Dako Corporation, Carpinteria, CA). For staining, deparaffinized and rehydrated sections were subjected to antigen retrieval for 10 min in Antigen Unmasking solution (Vector Laboratories, Burlingame, CA) at 100°C, and, after cooling, endogenous peroxidase activity was quenched with 3% H_2_O_2_ in methanol, followed by blocking in 20% goat serum in TPBS (0.01% Triton X-100 in PBS) before incubation with primary antibody in TPBS. For immunohistochemistry with detection using diaminobenzidine as substrate, slides were rinsed and subsequently treated with biotin-conjugated secondary antibodies (1:200) (Vector Laboratories) for 30 min at room temperature. To visualize biotin staining, the Vectastain Elite ABC kit was used (Vector Laboratories), followed by counterstaining with hematoxylin. For immunofluorescence analysis, Texas Red-goat antimouse (1:400), FITC-donkey antirabbit (1:100) and Cy3-donkey antimouse (1:200) secondary antibodies were used (Molecular Probes, Eugene, OR). ProLong Gold Antifade reagent with DAPI (Invitrogen) was used for nuclear counterstain and mounting medium. Control experiments were performed by incubating the slides in the presence of the secondary antibodies without the primary antibodies (data not shown).

In some experiments the slides were blocked as described above and stained for 1.5 h at 37°C with Alexa 488-conjugated Griffonia (Bandeiraea) simplicifolia lectin II (GS II) (1:1000). Paraffin sections were also stained with hematoxylin and eosin (H&E) to visualize cellular morphology, and periodic acid-Schiff (PAS)/Alcian blue stain was used to visualize neutral (pink) or acidic (blue) mucin, respectively (Shinohara et al. 2010). The H&E-stained paraffin sections were analyzed blindly by a comparative pathologist for epithelial cell changes in the oxyntic mucosa. Images were obtained on a Nikon Eclipse E800 microscope (Nikon, Melville, NY) using a Spot CD camera. Cell proliferation was determined by counting the number of cells with PCNA-positive nuclei in at least 10 glands of sections stained with anti-PCNA antibodies obtained from three to four mice from each group (Shinohara et al. 2010).

### Data analysis

Data are expressed as means ± SE. Statistical analysis was performed using Student’s *t*-test. *P* values < 0.05 were considered to be significant.

## Results

**Figure 1:**
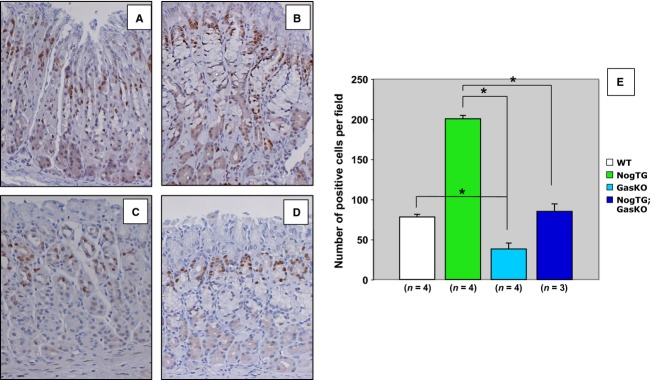
Regulation of gastric epithelial cell proliferation by gastrin in noggin TG-mice. Gastric paraffin sections from 12-week-old wild type (WT) (A), noggin TG (NogTG) (B), gastrin knock out (GasKO) (C), and NogTG;GasKO (D) mice were stained with antiproliferating cell nuclear antigen (PCNA) antibodies. Graph bars represent the number of PCNA-positive nuclei detected in the different groups of mice. Values are shown as means ± SE. *P < 0.05. Numbers in parenthesis indicate the number of separate mice that were used for the studies.

As hypergastrinemia stimulates gastric epithelial cell proliferation, we investigated if gastrin mediates the hyperproliferative response observed in the NogTG mice. The NogTG mice were crossed to GasKO mice to generate NogTG;GasKO mice. As shown in Figure1A and C, the GasKO mice exhibited a decrease in the number of PCNA-positive nuclei, when compared to WT mice. In addition, in agreement with our previously published reports (Shinohara et al. 2010; Takabayashi et al. 2014), inhibition of BMP signaling led to a significant increase in the number of proliferating cells, an effect that was lost in the NogTG;GasKO mice (Fig.1B and D). To confirm these observations, we counted the number of PCNA-positive nuclei in the fundic glands. As shown in the bar graphs (Fig.1E), lack of gastrin caused an almost complete loss of the hyperproliferative response observed in the gastric mucosa of the NogTG mice.

**Figure 2:**
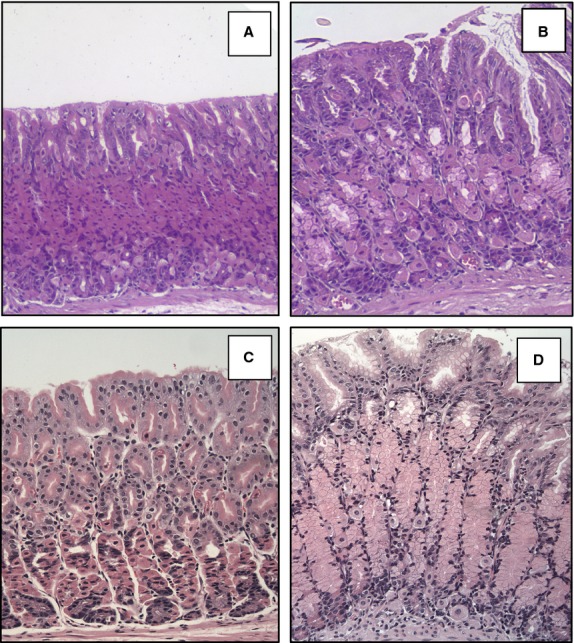
Development of morphological abnormalities in the gastric epithelium of NogTG;GasKO mice. Representative H&E-stained gastric paraffin sections of the corpus of 12-week old wild type (WT) (A), NogTG (B), GasKO (C), and NogTG;GasKO mice (D). Similar histological changes were observed in at least three other mice from each group.

To investigate the morphological changes induced by both inhibition of BMP signaling and lack of gastrin, we examined H&E-stained sections of the gastric mucosa obtained from WT, NogTG, GasKO, and NogTG;GasKO mice. While the fundic epithelium of the GasKO mice did not show any striking morphological alterations compared to that of WT, age matched littermates (Fig.2A and C), the mucosa of the NogTG;GasKO mice (Fig.2D) demonstrated the presence of an increase in the number of mucus-type cells, and a more dramatic reduction in the number of cells that had the morphological appearance of PCs, when compared to NogTG mice (Fig.2B).

**Figure 3:**
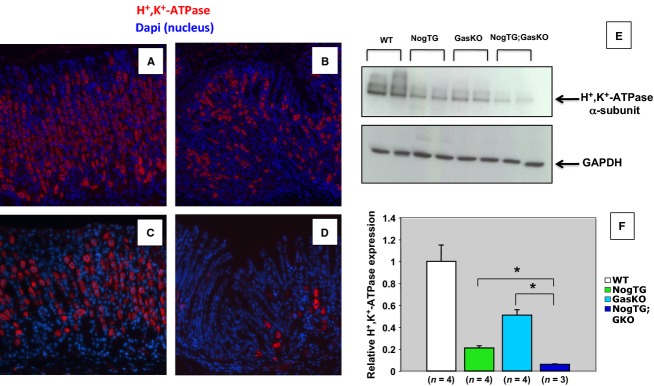
Regulation of parietal-cell number by gastrin and BMP signaling. Gastric paraffin sections from 12-week-old wild type (WT) (A), noggin TG (NogTG) (B), gastrin knock out (GasKO) (C), and NogTG;GasKO (D) mice were stained with the anti-H^+^,K^+^-ATPase *α*-subunit antibody and a CY-5-conjugated secondary antibody. Similar results were observed in at least three other mice from each group. H^+^,K^+^-ATPase *α*-subunit protein expression was measured by western blots (E). Each treatment group represents samples obtained from two separate mice. Identical results were observed in one additional mouse. H^+^,K^+^-ATPase *α*-subunit mRNA abundance was quantitated using QRT-PCR and it was displayed as fold-change over WT negative controls (F). Values are shown as means ± SE. *P < 0.05. Numbers in parenthesis indicate the number of separate mice that were used for the studies.

To better define these cellular features, we stained the sections with anti-H^+^,K^+^-ATPase *α*-subunit antibodies to detect PCs. As shown in Figure3, while the GasKO mice did not show any major abnormalities when compared to WT controls (Fig.3A and C), the NogTG;GasKO mice (Fig.3D) exhibited a reduction in the number of H^+^,K^+^-ATPase-positive cells which was more prominent than that seen in the NogTG mice (Fig.3B). These observations were further validated by western blots (Fig.3E) and QRT-PCR assays (Fig.3F).

**Figure 4:**
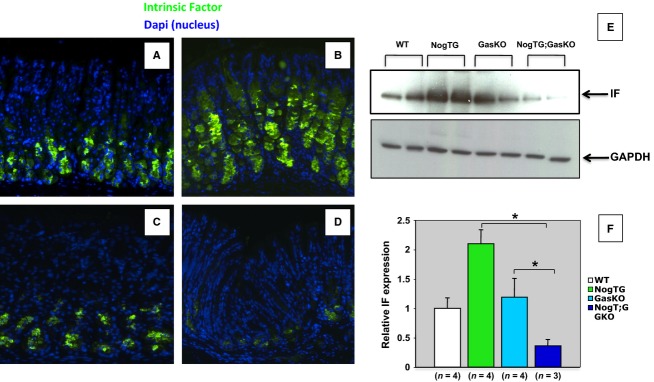
Regulation of zymogenic cell markers by gastrin and BMP signaling. Gastric paraffin sections from 12-week-old wild type (WT) (A), noggin TG (NogTG) (B), gastrin knock out (GasKO) (C), and NogTG;GasKO (D) mice were stained with the anti-IF antibody and a FITC-conjugated secondary antibody. Similar results were observed in at least three other mice from each group. IF protein expression was measured by western blots (E). Each treatment group represents samples obtained from two separate mice. Identical results were observed in one additional mouse. IF mRNA abundance was quantitated using QRT-PCR and it was displayed as fold-change over WT negative controls (F). Values are shown as means ± SE. *P < 0.05. Numbers in parenthesis indicate the number of separate mice that were used for the studies.

To investigate the role of gastrin and BMP signaling in ZC differentiation we stained the sections with specific anti-IF antibody antibodies. While the GasKO mice did not show any major abnormalities in IF staining when compared to WT controls (Fig.4A and C), the NogTG mice exhibited, in agreement with our previously published reports (Shinohara et al. 2010), an increase in the number of IF-positive cells (Fig.4B), an effect that was lost in the NogTG;GasKO mice (Fig.4D). Western blots (Fig.4E) and QRT-PCR assays (Fig.4F), indicated that the GasKO mice do not exhibit significant changes in IF expression. In contrast, while the NogTG mice showed increased expression of both the IF protein and gene, the NogTG;GasKO did not, demonstrating the presence of a significant decrease in the expression of IF when compared to WT, NogTG, and GasKO mice.

**Figure 5:**
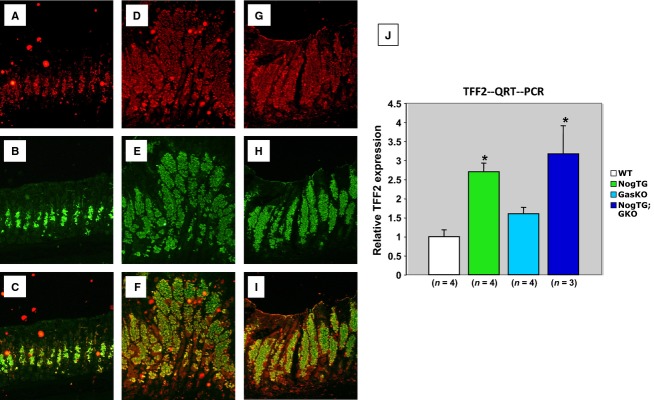
Regulation of mucus neck cell markers by gastrin and BMP signaling. Gastric paraffin sections from 12-week-old WT (A–C), NogTG mice (D–F), and NogTG;GasKO mice (G–I) were stained with anti-TFF2 primary antibodies and texas red-conjugated secondary antibodies (red) together with Alexa 488-conjugated GS II (green). Merged images are shown in C, F, and I. Similar results were observed in one other mouse from each group. TFF2 mRNA abundance was measured using QRT-PCR and it was displayed as fold-change over WT negative controls (J). Values are shown as means ± SE. *P < 0.05. Numbers in parenthesis indicate the number of separate mice that were used for the studies.

Trefoil factor 2 (TFF2), is a peptide growth factor that, in the normal stomach, is expressed in deep gland cells of the antrum and in the mucus neck cells of the fundic glands (Goldenring et al. 2000; Nomura et al. 2004a; Goldenring and Nomura 2006). Moreover, aberrant expansion of TFF2 expression to the base of the corpus glands has been associated with the occurrence of spasmolytic polypeptide expressing metaplasia (SPEM), an event linked to the development of both dysplasia and neoplasia (Goldenring et al. 2000; Nomura et al. 2004a; Goldenring and Nomura 2006). SPEM cells are also positively stained with the lectin GSII, which, in the normal stomach, binds to mucus neck cell mucins (Shinohara et al. 2010). In previously published studies we reported that inhibition of BMP signaling in the stomach of mice leads to SPEM, as we noted increased TTF2 mRNA and protein expression and expansion of cells, at the bottom of the fundic glands, that could be positively stained with GSII (Shinohara et al. 2010; Takabayashi et al. 2014). In this study, we did not detect any significant changes in both TFF2 and GS-II staining between WT (Fig.5A–C) and GasKO mice (data not shown). As previously reported (Shinohara et al. 2010; Takabayashi et al. 2014), we noted a marked increase in the number of TFF2- and GS-II-positive cells (Fig.5D–F) in the NogTG mice. This phenotype was not affected by lack of gastrin, as both NogTG mice and NogTG;GasKO mice demonstrated similar patterns of TFF2 and GSII staining (Fig.5G–I). Similarly, the increased level of expression of TFF2 mRNA that was observed in the NogTG mice was not affected by lack of gastrin (Fig.5J). These findings suggest that gastrin is not involved in the development of SPEM in mice expressing an inhibitor of BMP signaling in the gastric epithelium.

## Discussion

In this manuscript we report a series of novel observations that underscore the importance of gastrin and the BMPs in the regulation of gastric epithelial cell proliferation and differentiation. In particular, we present evidence that inhibition of BMP signaling and loss of gastrin leads to a series of abnormalities in the normal mechanisms that regulate the proliferation of the gastric epithelium and the differentiation of parietal and ZCs.

Our study shows that gastrin mediates, the hyperproliferative state observed in the epithelium of the Noggin transgenic mice. This effect is consistent with the observation that gastrin stimulates gastric epithelial cell growth through the induction of polypeptide growth factors such as heparin-binding EGF and amphiregulin, and the activation of enzymes, such as the ERKs (Dockray 1999; Yassin 1999), that exert well-established growth-promoting effects on both normal and neoplastic gastrointestinal tissues.

Several studies have demonstrated that the PCs play an important role in the process of differentiation and development of other cell lineages in the gastric mucosa, as loss of mature PCs alters the normal differentiation program of the gastric epithelium and causes different types of mucosal cell remodeling (Li et al. 1995, 1996; Canfield et al. 1996; Lopez-Diaz et al. 2006; Jain et al. 2008). In our previous investigations we reported that inhibition of BMP signaling in the stomach leads to a marked decrease in the number of PCs and to the appearance of aberrant lineages that express markers of both zymogenic and mucus neck cell differentiation (Shinohara et al. 2010; Takabayashi et al. 2014). In this report we observed that gastrin cooperates with the BMPs to regulate the process of parietal maturation and differentiation, as loss of gastrin enhanced the phenotypic abnormalities seen in the PCs of the noggin transgenic mice. These findings appear to be consistent with the observation that the PCs of the gastrin knockout mice are immature and less differentiated, demonstrating reduced expression of gene markers of differentiated function and upregulation of genes, such as those regulated by Wnt, that are characteristic of poorly differentiated epithelial cells (Jain and Samuelson 2006; Jain et al. 2006). As the BMP signal transduction pathway is known to negatively regulate the expression of Wnt-target genes (He et al. 2004), it is possible that loss of both BMP signaling and gastrin might cause an additive and/or potentiating increase in Wnt signaling, which could lead to an arrest in the normal programs of PC maturation and differentiation.

Several studies have suggested that in mice, the ZCs differentiate from pluripotent stem cells through a multi-step process that involves the progressive transition of mucus neck cells into fully differentiated ZCs (Nam et al. 2010). It has been also shown that this process might be regulated by parietal-cell secreted factors as loss of PCs leads to alterations in the normal process of ZC maturation and differentiation (Li et al. 1995, 1996; Canfield et al. 1996; Lopez-Diaz et al. 2006; Jain et al. 2008; Shinohara et al. 2010; Takabayashi et al. 2014). It is therefore possible that the abnormalities in ZC differentiation that we observed in the NogTG;GasKO mice might be mostly secondary to the presence of aberrant, poorly differentiated PCs, although we cannot completely exclude that both gastrin and the BMPs might have cooperative, direct effects on the ZCs.

Recent evidence has suggested that the ZCs can dedifferentiate during different pathological conditions of the gastric epithelium, giving rise to SPEM (Nam et al. 2010). Interestingly, although we noted robust occurrence of SPEM in the Noggin transgenic mice, loss of gastrin did not significantly affect the development of SPEM caused by inhibition of BMP signaling, suggesting that, gastrin does not participate in the development of this type of metaplasia. These data are in agreement with previously published reports indicating that SPEM develops in response to PC injury caused by the protonophore DMP-777, independent of gastrin (Nomura et al. 2004b). Thus, it appears to be a divergence between gastrin and BMP signaling regarding the control of zymogenic progenitors and the occurrence of SPEM, as the BMPs but not gastrin appear to control the process of ZC transdifferentiation that leads to the aberrant expression of TFF2.

In summary, we show that loss of BMP signaling and lack of gastrin lead to altered maturation and differentiation of zymogenic and PC lineages. Gastrin deficiency also induces loss of the hyperproliferative response of the gastric epithelium induced by inhibition of BMP signaling but it does not seem to affect the expression of markers of SPEM.

These findings shed new insight into the complex signal transduction pathways that mediate the actions of growth factors in the stomach, providing new clues for a better understanding of the mechanisms that regulate gastric epithelial cell growth and differentiation.
